# Anomalous and Not-So-Common Behavior in Common Ionic Liquids and Ionic Liquid-Containing Systems

**DOI:** 10.3389/fchem.2019.00450

**Published:** 2019-06-20

**Authors:** José M. S. S. Esperança, Mohammad Tariq, Ana B. Pereiro, João M. M. Araújo, Kenneth R. Seddon, Luis Paulo N. Rebelo

**Affiliations:** ^1^LAQV/REQUIMTE, Faculdade de Ciências e Tecnologia da Universidade Nova de Lisboa, Caparica, Portugal; ^2^QUILL Research Centre, the Queen's University of Belfast, Belfast, United Kingdom

**Keywords:** ionic liquids, unusual behavior, thermal expansion coefficient, viscosity, surface tension, odd-even effects, reversed charge ILs, LCST

## Abstract

This work highlights unexpected, not so well known responses of ionic liquids and ionic liquid-containing systems, which are reported in a collective manner, as a short review. Examples include: (i) Minima in the temperature dependence of the isobaric thermal expansion coefficient of some ILs; (ii) Viscosity Minima in binary mixtures of IL + Molecular solvents; (iii) Anomalies in the surface tension within a family of ILs; (iv) The constancy among IL substitution of C_p_/V_m_ at and around room temperature; (v) ILs as glass forming liquids; (vi) Alternate odd-even side alkyl chain length effects; (vii) Absolute negative pressures in ILs and IL-containing systems; (viii) Reversed-charged ionic liquid pairs; (ix) LCST immiscibility behavior in IL + solvent systems.

## Introduction

Ionic Liquids (ILs) are constituted quasi-exclusively by anions and cations, melting at temperatures that are much lower than those of their conventional, inorganic salts counterparts. For instance, “table salt” (NaCl) melts at about 800°C. In contrast, many ILs present melting points lower than room temperature. Generally, ILs have a large liquid range and, some of them do not easily crystallize on cooling, instead they supercool and undergo a glass transition.

Several high quality reviews (Welton, 1999, 2018; Plechkova and Seddon, 2008; Armand et al., 2009; Hallett and Welton, 2011; Niedermeyer et al., 2012; Tariq et al., 2012; Chatel et al., 2014; Hayes et al., 2015; Hunt et al., 2015; Podgoršek et al., 2016; Kar et al., 2019) are available. No review focused on the unexpected behavior of ILs has been published, where a scrutiny of their not so well known physical chemistry responses is made. This work constitutes a first attempt in this direction.

With the upsurge in the research activity around these novel salts during the last two decades, peculiar, unique and interesting behavior of these complex materials have been revealed. ILs are considered a link between molten salts and molecular solvents (Leal et al., 2009).

Lopes and Pádua (2006) using MD simulations and Triolo et al. (2007) using experimental X-ray data have demonstrated that ILs, even in their pure state, contain nanostructured organization at a molecular level. In addition, this very distinct feature has been confirmed using experimental thermodynamic approaches (Pereiro et al., 2013; Rocha et al., 2013). This phenomenon is responsible for many of their peculiar behavior. Also, how a particular IL interacts with the co-solvent is very unique and depends on the interactions between the IL and the chemical nature of the other component: polar/apolar/associated fluids (Lopes et al., 2006; Pádua et al., 2007).

The topics tackled in this contribution are not comprehensive. Examples not herein presented include: their ability to form halogen-bonds (Bernardes and Canongia Lopes, 2017; Saccone et al., 2017; Cavallo et al., 2018; Lodeiro et al., 2018), the formation of liquid crystals (Alvarez Fernandez and Kouwer, 2016; Goossens et al., 2016), the total miscibility in water of fluorinated ILs (Pereiro et al., 2015; de Ferro et al., 2018), and other unexpected behavior in respect to their physical properties (Singh et al., 2015, 2017; Dzida et al., 2018; Rahman and Senapati, 2018). Zwitterionic liquids (Blesic et al., 2017; Ohno et al., 2018; Wu et al., 2018), which fill the gap between small-ion ILs and ILs with strong H-bonds, as well as hydrated ILs (Haberler et al., 2012; Fujita et al., 2016), where H-bonds between one or both of the ions and a small number of water molecules is sufficient to produce new materials with superior properties, constitute other examples of not so-well-known behavior.

## Minima in the Temperature Dependence of the Isobaric Thermal Expansion Coefficient of Some ILs

For most liquids the isobaric thermal expansion coefficient, α_p_, is positive and increases with increasing temperature as it has to diverge to a positive infinite value at the critical point. The best well-known example of a distinct behavior is that of water, in which α_p_ at about atmospheric pressure is negative at temperatures between 0 and 3.98°C, meaning a contraction on heating. At the temperature of maximum density, 3.98°C, α_p_ reaches a null value, becoming positive for higher temperatures. Despite this “anomaly,” the α_p_ of water always increases with increasing temperature. In this section, we highlight examples of the anomalous behavior of α_p_ with temperature increase for some ionic liquids. The results discussed in this section are the first examples of substances that show a minimum in α_p_ as temperature increases.

Conflicting results were reported earlier about the temperature dependence of α_p_ of ILs (Rebelo et al., 2004). Later some reports for a small set of ILs have shown that it is possible to obtain negative values for (∂α_p_/∂T)_p_ (Rodríguez and Brennecke, 2006; Sanmamed et al., 2007). Accurate determinations of this property may be difficult since the determination of α_p_ from the temperature dependence of density, ρ(T), can produce numerical artifacts (Cerdeirina et al., 2001; Troncoso et al., 2010). Most often the density of ILs is measured using a vibrating tube densimeter. Their high viscosity may influence the determination of the density and, if viscosity corrections are not taken into consideration, erroneous α_p_(T) values may be obtained (Sanmamed et al., 2007).

Systematic studies on the measurements of density along a homologous series and wide temperature and pressure conditions are not commonly reported. In order to fill this gap, the densities of a series of ILs in a wide temperature and pressure range were reported. Tariq et al. (2010) have measured the densities of imidazolium bistriflimide ILs, [C_n_C_1_im][Ntf_2_], where *n* = 2–14 in the temperature range of 283–483 K. This dataset was the largest one both in terms of an extended homologous series of ILs, as well as in a large temperature interval, enabling the authors to study subtle specificities of their thermal response. Nieto de Castro et al. (2010) have carried out high-precision density measurements over broad temperature (293–473 K) and pressure (0.1–60 MPa) ranges on four ILs, namely [C_4_C_1_im][Ntf_2_], [C_4_C_1_im][dca], [C_2_C_1_im][C_2_SO_4_], and [Aliquat][dca].

**Figure 1:**
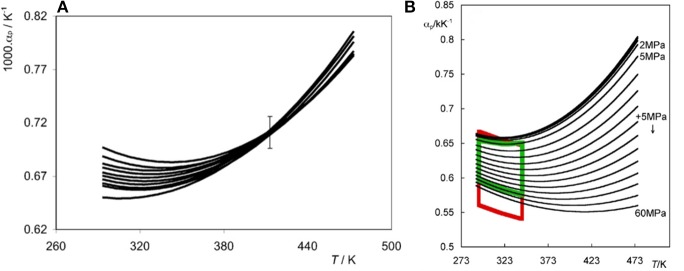
**(A)** Thermal expansion coefficient, α_p_ as a function of temperature and at atmospheric pressure for each member of the [C_n_C_1_im][Ntf_2_] family (C_14_-C_2_ from top to bottom). The error bar corresponds to an average uncertainty for all fits. (Reprinted from Tariq et al., 2010 with permission from Elsevier) **(B)** α_p_ for [C_4_C_1_im][Ntf_2_] as a function of temperature at different pressures. The top four unlabeled isobars in the right panel correspond to pressures of 0.1, 0.25, 0.5, and 1 MPa. The red lines represent the boundaries of the data presented by Navia et al. (2010a), Navia et al. (2010b) (isotherms between 293 and 353 K and isobars between 5 and 50 MPa). The green lines highlight the same boundaries for the set of data measured by Nieto de Castro et al. (2010). (Reprinted from Nieto de Castro et al., 2010 with permission from Elsevier).

It has been demonstrated (Tariq et al., 2010) that in the [C_n_C_1_im][Ntf_2_] series, an increase in temperature results on a small decrease in the values of α_p_. This inverse proportionality between α_p_ and T constitutes an anomalous behavior and is observed at “low-temperature.” Since thermodynamics impose that at the liquid–vapor critical point, a divergence to +∞ in α_p_ ought to occur, at some lower temperature a minimum in α_p_ = f(T) is obtained (Figure 1A).

Similarly, densities of four ILs in wide pressure and temperature ranges were measured (Nieto de Castro et al., 2010). Again, α_p_ decreases as temperature increases in the low-T range (Figure 1B). Navia et al. (2010a), Navia et al. (2010b) also obtained data for a large set of ILs confirming negative (∂α_p_/∂T)_p_ values at low temperatures.

These works have shown that the temperature derivative of the thermal expansion coefficient, (∂α_p_/∂T)_p_, of some ILs changes sign at a temperature that depends both on pressure and IL nature.

## Viscosity Minima in Binary Mixtures of IL + Molecular Solvents (MSs)

Most models for the viscosity of a mixture predict that the mixture's viscosity of two components with identical viscosities is invariant along the whole composition range.

A minimum in viscosity-composition plots of binary mixtures is an unusual phenomenon, which has been observed for some non-polar + polar systems (Kouris and Panaylotou, 1989; Papanastaaiou and Ziogas, 1991; Laesecke et al., 2007). However, the molecular reasoning behind this phenomenon is not very well understood (Srinivas et al., 2001; Abraham et al., 2007) and thereby it is hard to model the viscosity values of such mixtures using existing mixing rules and predictive methods (Qunfang and Yu-Chun, 1999). Such uncommon phenomenon has been shown for the first time in systems containing ionic liquids (ILs) + molecular solvents (MSs) (Tariq et al., 2015).

**Figure 2:**
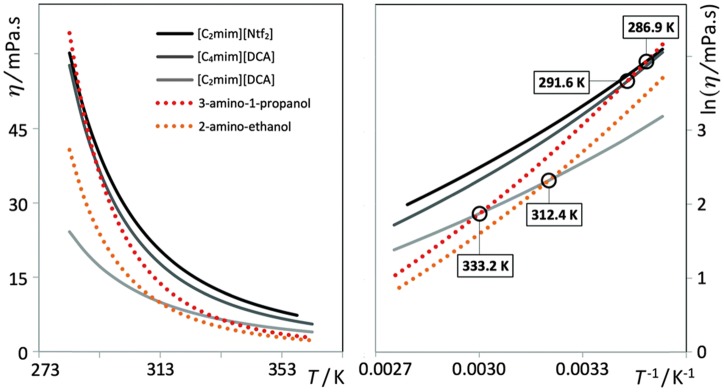
Experimental viscosity data, η/mPa·s, of the three ILs ([C_2_mim][Ntf_2_], [C_2_mim][DCA], and [C_4_mim][DCA]) and two molecular solvents (2AE and 3AP) studied in this work as a function of temperature. The right-hand plot shows the logarithm of viscosity as a function of the reciprocal of temperature. The circles indicate the viscosity cross-over temperatures. (Reprinted from Tariq et al., 2015 with permission from Royal Society of Chemistry).

Tariq et al. (2015) selected four binary IL+MS systems composed of a molecular solvent (2-amino-ethanol (2AE) or 3-amino-1-propanol (3AP)) and an IL (from the 1-alkyl- 3-methylimidazolium family ([C_n_mim]^+^) using dicyanamide ([DCA]) or bistriflimide ([Ntf_2_]) as the anion). All the components forming the four binary systems are completely miscible in the entire composition range and show crossover temperatures where the IL and MS viscosity values are identical (Figure 2).

**Figure 3:**
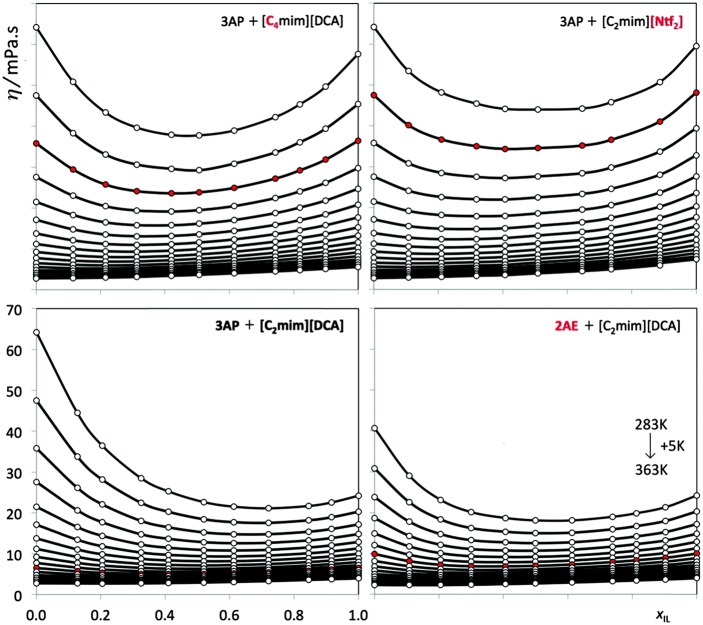
Experimental viscosity data, η/mPa·s, of four (ionic liquid plus molecular solvent) binary mixtures as a function of the ionic liquid mole fraction, x_IL_. The curves represent different isotherms. The red circles denote for each system the isotherm closest to the viscosity crossover temperature. (Reprinted from Tariq et al., 2015 with permission from Royal Society of Chemistry).

The η(T,x) plots presented in Figure 3 reveal that (i) there is a viscosity minimum at low temperatures for IL-rich mixtures and that (ii) the viscosity minimum is centered around the equimolar composition as one approaches the temperature at which both components present identical viscosities. The overall viscosity trend for these systems is similar along the composition/temperature surface.

The origin of the viscosity minima is 2-fold: (i) it comes from the different cohesive energies of both pure MSs and ILs and (ii) from changes in the structure and interactions of the mixtures compared to the pure components.

The molecular interactions between the molecules can be revealed by comparison of the cohesive energies of the two classes of components (obtained through vaporization enthalpies at room temperature). The values of the vaporization enthalpies of the MSs are in the range of 60–70 kJ mol^−1^ (Marsh et al., 2004; Yaws, 2009; Acree and Chickos, 2010). In contrast, ILs present vaporization enthalpies above 135 kJ mol^−1^ (Marsh et al., 2004; Esperança et al., 2010). In the case of these MSs, the cohesive energy is largely related to the intra-hydrogen bonding between the functional groups of each molecule. In the case of ILs, MD simulations (Santos et al., 2007; Shimizu et al., 2010) have revealed that Coulomb forces play an important role, contributing substantially for their enhanced cohesive energy.

H-bonds between the different ILs and MSs and the structural differences between the mixture and the pure molecular components originates a reduction on the viscosity of the mixtures. Other type of uncommon behavior of IL containing binary mixtures has been reported by Andrzejewska et al. (2009) and Trivedi and Pandey (2011), where a maximum in the viscosity has been found in mixtures of IL + polymers. It should be noted that systems containing polymers are known to show non-Newtonian behavior.

## Anomalies in the Surface Tension Within a Family of ILs

**Figure 4:**
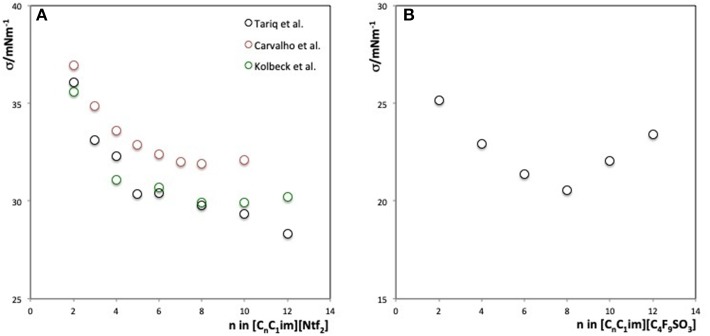
**(A)** Surface tension values vs. the size of the alkyl chain, *n*, for [C_n_C_1_im][Ntf_2_] family from different authors measured at 293 K showing clearly a break around C_5_-C_6_. **(B)** Surface tension values as a function of the alkyl chain, *n*, for the [C_n_C_1_im][C_4_F_9_SO_3_] family at 313 K. (Reprinted with permission from Luís et al., 2016. Copyright (2016) American Chemical Society).

The values of the surface tension of most ILs fall in between those of water and molecular solvents. One interesting trend has been found in the 1-alkyl-3-methylimidazolium bistriflimide, [C_n_C_1_im][Ntf_2_] family (Carvalho et al., 2008; Kolbeck et al., 2010; Tariq et al., 2010; Haddad et al., 2018; Shimizu et al., 2018). Carvalho et al. (2008) were the first to report that the surface tension values of [C_n_C_1_im][Ntf_2_] series do not decrease linearly with the alkyl chain length increase (*n* = 2–10), but rather reach a plateau for long cation side alkyl chains. Later, Tariq et al. (2010) measured the surface tension for C_2_-C_14_ within a broad temperature range of 303–493 K and Kolbeck et al. (2010) measured it at room temperature for C_1_-C_12_. Both confirmed these trends (Figure 4A).

The trend is not regular, and was checked out by three distinct works: there is a substantial decrease in the surface tension value from [C_2_C_1_im][Ntf_2_] to [C_5_C_1_im][Ntf_2_] and a relatively lower decrease from [C_6_C_1_im][Ntf_2_] to [C_12_C_1_im][Ntf_2_]. Changes in the ratio of prominence of non-polar to polar moieties of the ILs at the surface are a consequence of the length of the alkyl side chains. MD simulations and X-ray diffraction studies (Lopes and Pádua, 2006; Triolo et al., 2007) have shown the creation of a second nanostructured domain (formation of non-polar continuous domains) for ILs with alkyl chain length equal or >6 carbon atoms.

Recently, Shimizu et al. (2018) have been able to predict the surface tension of the 1-alkyl-3-methylimidazolium bistriflimide family by combining angle-resolved X-ray photoelectron spectroscopy data and MD simulations results using the Langmuir principle.

Haddad et al. (2018) have also used angstrom-resolution X-ray methods to understand the reason behind the peculiar behavior of the surface tension within the [C_n_C_1_im][Ntf_2_] homologous series. They also found a distinct behavior for ILs with alkyl chain length equal or longer than six carbon atoms due to the formation of nanosegregated domains (polar/apolar) which create alternating layers at the surface. This study clarifies the liquid–air interface structure for a common homologous series of ILs. By varying the cation's alkyl chain length one can tune the interactions's importance, from long-range coulombic forces to short-range van der Waals interactions. Such variation causes the interface structure to turn from simple, to layered, to liquid crystalline. The quantitative results obtained from this work may constitute a reference for validating simulations and theory.

An even more peculiar behavior of the surface tension within a homologous series has been reported by Luís et al. (2016) for [C_n_C_1_im] cation based ILs (where *n* = 2–12) combined with perfluorobutanesulfonate anions. Instead of a plateau, a minimum in the surface tension is observed for a cation alkyl side chain of 8 (Figure 4B). The existence of three nanosegregated domains (polar, apolar, and fluorinated) for the long cation's alkyl chain length is the main reason for this distinctive trend. More specifically, it results from the competition between the diverse domains for the gas-liquid interface. Their surface entropy is the lowest when compared to conventional ILs.

## The Constancy Among IL Substitution of C_p_/V_m_ at and Around Room Temperature

The temperature dependence of the enthalpy is known as heat capacity. Some heat capacity changes may indicate the occurrence of a phase transition and enable to understand variations in the structure of the compounds.

Zabransky et al. (1990) and Domalski and Hearing (1996) compiled and analyzed, independently, the heat capacity data for a huge number of substances. To the best of our knowledge, group contribution methods and/or corresponding state principal based methods are the approaches generally used to estimate liquid heat capacities.

**Figure 5:**
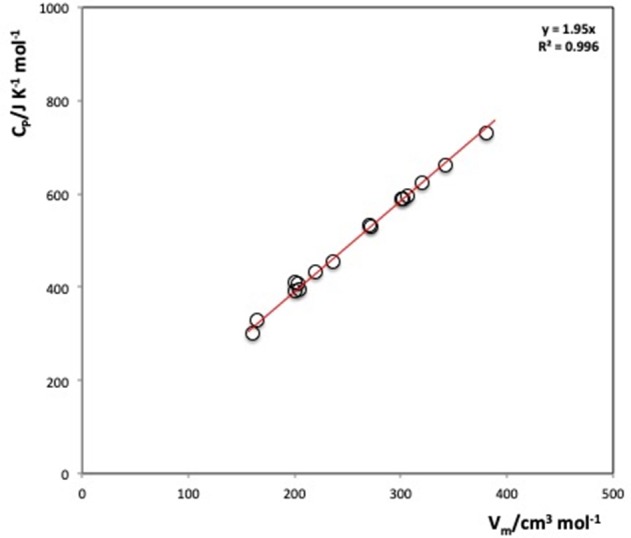
IL heat capacity as a function of the molar volume (*T* = 298.15 K). [Drawn using the data presented in Paulechka et al. (2010)].

For ionic liquids, Strechan et al. (2008) and Gardas and Coutinho (2008) demonstrated independently that there is a linear relationship between the heat capacities and the molar volumes of imidazolum, pyridinium, and pyrrolidinium based-ILs (Figure 5). They have used the dataset of limited number of ILs to establish this correlation and propose that the volumetric heat capacity of ILs is almost invariant at 298.15 K.

Later, Paulechka et al. (2010) have refined this relation by carefully selecting dataset of 19 ILs (for which the precise density data was available) and measured their heat capacity with an uncertainty of (±1%). They have also witnessed that there is constancy among the volumetric heat capacities of ILs. The average value at 298.15 K is:

(1) $$\begin{array}{l} {\text{C}}_{\text{p}}/{\text{V}}_{\text{m}}=1.95\pm 0.02\;\left(\text{J}{\text{K}}^{-1}{\text{cm}}^{-3}\right) \end{array}$$

This average value agrees well with those of Gardas and Coutinho (2008). The difference between the experimental volumetric heat capacities and this C_p_/V_m_ value was found to be < ±5%. Contrarily to molecular solvents, a simple rule exists to predict the heat capacity of distinct ILs.

It has been demonstrated that C_p_/V_m_ is basically independent of the structure of the IL. Therefore, Paulechka et al. (2010) concluded that ILs used for application as heat accumulators should be chosen on the basis of other parameters such as thermal stability, viscosity, thermal conductivity, to name a few.

## ILs as Glass Forming Materials

ILs have low melting points (Tm) when compared to conventional inorganic salts. This fact gives them the status of a unique class of ionic materials composed almost solely of cations and anions that exist in the liquid state at or near room-temperature. Many ILs that do exist in the liquid state never crystallize and thus do not show any melting point, instead, on cooling they show a glass transition (Tg). Most ILs (Valderrama et al., 2017) show a glass transition temperature in the 150–250 K range.

It is easier to accurately determine melting points (typically for ILs a good (±5 K) uncertainty is obtained) than those of vitrification—large discrepancies (up to 20%) have been found in reported Tg values. Glass transitions do not occur at a specified fixed temperature as they are not first-order changes (Brandrup et al., 1999), and are kinetically dependent. The dependence of Tg of ILs on the scan rate has thoroughly been investigated (Goḿez et al., 2013; Tao et al., 2017).

**Figure 6:**
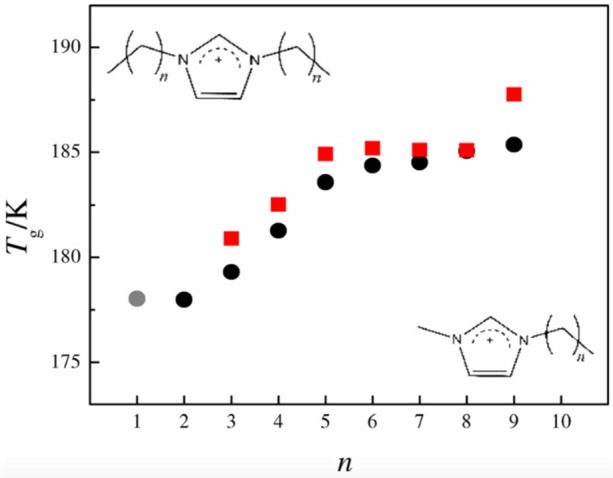
Glass transition temperatures (T_g_) for the [C_n_C_1_im][Ntf_2_] (black circles) and [C_n_C_n_im][Ntf_2_] (red squares) series as a function of n (number of carbon atoms in one alkyl side chain); [C_1_C_1_im][Ntf_2_] (gray circle). (Reprinted from (Rodrigues and Santos, 2016) with permission from John Wiley and Sons).

Blokhin et al. (2006) and Rodrigues and Santos (2016) have continuously presented high quality thermal properties data of ILs and the latter demonstrated how the nanostructuration of [C_n_C_1_im][NTf_2_] and [C_n_C_n_im][NTf_2_] family influences their glass and melting temperatures. The work demonstrates a variation of the Tg behavior at the critical alkyl size, when *n* = 6 (Figure 6). T_g_ increases as the alkyl side chain of the imidazolium cation grows, mainly due to the enhancement of the van der Waals interactions. For alkyl chains longer than *n* = 6, a plateau in the Tg values after nano-structuration is observed.

Some works have tackled the prediction of the Tg of ILs (Mirkhani et al., 2012; Valderrama et al., 2017). Other studies have identified difficulties behind observing IL's crystallization (Serra et al., 2017; Ferreira et al., 2019).

Lima et al. (2018) have measured the Tg and Tm of a pyrrolodinium based-IL, [C_4_C_1_Pyrr][Ntf_2_], from atmospheric pressure up to an extremely high pressure of 2 GPa using X-ray diffraction and Raman scattering techniques. They have found that Tg and Tm both follow similar pressure dependences.

**Figure 7:**
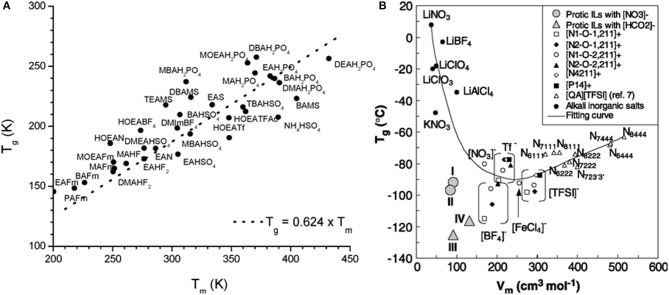
**(A)** Melting points vs. glass transition temperatures for PILs reported by Belieres and Angell (2007). (Reprinted with permission from Belieres and Angell, 2007. Copyright (2007) American Chemical Society) **(B)** T_*g*_ values as a function of molar volume (V_m_) for PILs reported by Xu and Angell (2003). The line through the points is a guide to the eye. [From Xu and Angell (2003). Reprinted with permission from AAAS].

At atmospheric pressure, most compounds seem to follow the well-known “2/3 golden rule” meaning that the ratio of glass transition temperature (Tg) to that of melting (Tm), Tg/Tm, for all compounds should be around 0.66. Belieres and Angell (2007) have collected data for several protic ionic liquids (PILs) and showed that similar to other materials, most of the PILs fall in the 2/3 line (Figure 7A). However, there are outliers that can reach a ratio as high as 3/4. Xu and Angell (2003) have discussed the Tg dependence on the molar volume (Figure 7B). Recently, Ferreira et al. (2019) and Serra et al. (2017) have shown that many of the aprotic ILs show high Tg/Tm values that are close to the $\frac{3}{4}$ value.

## Alternate Odd-Even Side Alkyl Chain Length Effects

Whenever the thermophysical characterization of the members of a family of ILs within a homologous series is performed, odd members are often neglected (Tariq et al., 2009). The main difficulty in the synthesis of ILs with alkyl chains, C_n_, where n is an odd number, is the high cost of their chemical precursors.

**Figure 8:**
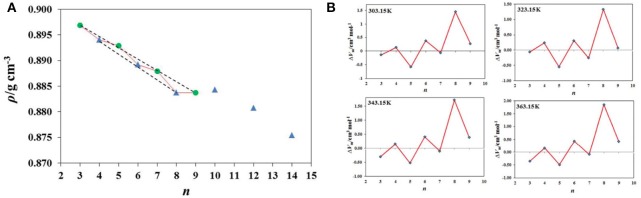
**(A)** Experimental densities of [P_888n_]Cl ionic liquids at 303.15 K, where *n* = 3,5,7, and 9 (green circles), *n* = 4,6,8,10,12, and 14 (blue triangles). (Reprinted from Adamová et al., 2011 with permission from Royal Society of Chemistry) **(B)** See-saw, even/odd alternation effect in the form of deviations (residuals) between experimental and fitted molar volume (V) data as a function of *n*, at four different temperatures. (Reprinted from Adamová et al., 2011 with permission from Royal Society of Chemistry).

Adamová et al. (2011) have measured the densities of a series of alkyltrioctylphosphonium chloride, [P8 8 8 *n*]Cl based ILs and found that the density values show a clear odd-even chain length alternation effect (Figure 8A). There are two independent trends: one for the odd and another one for the even-numbered compounds. This was observed up to *n* = 9. Data analyzed in terms of their molar volume, *V*m = *M*/ρ, presents remarkably this see-saw effect (Figure 8B).

Adamova et al. (2014) have carried out *Ab initio* calculations and MD simulations to understand the molecular reasoning behind these effects. Simulation runs revealed that the type of conformation/packing of the liquid (transoid conformations of the cation and head-to-head packing) are responsible for the observed alternation effects. The unexpected results first seen for the [P8 8 8 *n*]Cl series, enabled the authors to reveal a similar trend for another IL family, [C_n_C_1_im][NTf_2_]. Moreover, this see-saw effect, already known for the solid phase of linear alkanes and alkanols, was also seen in their liquid phase molar volume.

**Figure 9:**
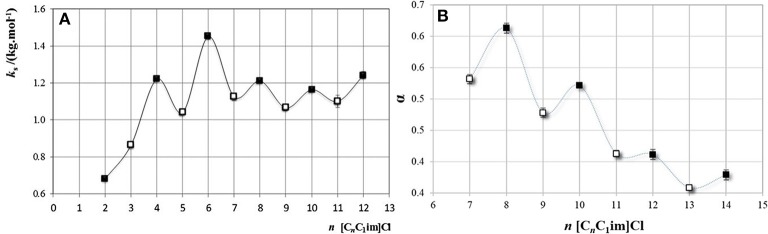
**(A)** Setschenow constant, *ks*, as a function of the carbon number at the longest alkyl chain, *n*, in [C_n_C_1_im]Cl, for the ABS formed with the salt K_2_CO_3_. (Reprinted from Belchior et al., 2018 with the permission of AIP Publishing) **(B)** Degree of ionization as a function of the carbon number at the cation alkyl chain, *n*, in [C_n_C_1_im]Cl. (Reprinted from Belchior et al., 2018 with the permission of AIP Publishing).

Very recently, it has been shown (Belchior et al., 2018) that diluted solutions of [C_n_C_1_im]Cl (*n* = 2–14) used to form aqueous biphasic systems (ABS) with salts also exhibit such odd/even effects. The odd/even effect was observed in the Setschenow salting-out coefficients (*ks*) for systems containing water, [C_n_C_1_im]Cl and K_2_CO_3_ (Figure 9A). The salting-out ability depends on the molar volume of the IL, and therefore the alternation odd-even volume pattern is reflected in the *ks* values. This is more prominently seen for ILs with alkyl side chain length up to *n* = 6. As for the CMC values of the [C_n_C_1_im]Cl series this effect was not observed. In contrast, it was also shown that an odd-even effect occurs for properties, such as, the degree of ionization (Figure 9B), the molar conductivity, and the molar conductivity at infinite dilution.

In addition to the above-discussed cases, odd/even effects have also been encountered in several other IL properties, such as, viscosities (Rocha et al., 2013), entropy and enthalpy of vaporization (Rocha et al., 2012, 2014), glass forming behavior (Leys et al., 2014), and diffusion coefficients (Yang et al., 2016).

## Absolute Negative Pressures in ILs and IL-containing Systems

Liquids can be mechanically stretched. If this is performed in an isotropic fashion it is possible to obtain absolute negative pressures. Most of the focus of negative pressure experiments was placed on water samples and only very recently some results for pure ionic liquids appeared. The results show that a variety of commonly used ionic liquids (ILs) can be stretched successfully to tensions of about −100 MPa in Pyrex glass capillaries of internal volume of ~0.05 cm^3^ (Silva et al., 2018). These results contrast with the ones obtained for water, in which the maximum stretching of samples of similar size was around −35 MPa (Visak et al., 2002, 2003).

The main reasons for achieving such enormous absolute negative pressures in ionic liquids appears to be a consequence of distinct properties, namely almost null volatility, increased viscosity compared to common liquids, low surface tension, enhanced wettability toward pyrex glass, and easiness to supercool.

**Figure 10:**
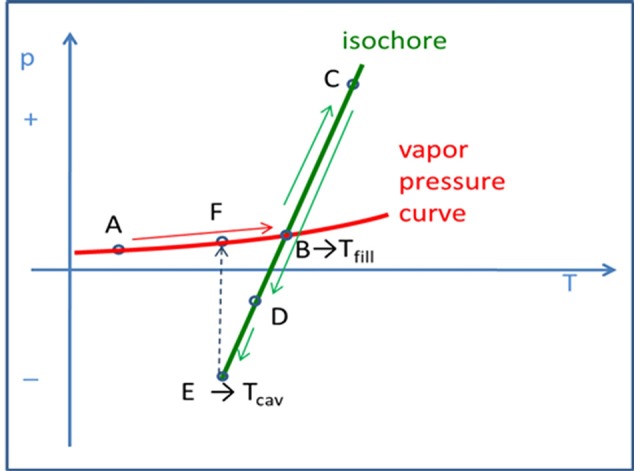
The Berthelot cycle used to obtain tensioning in the IL samples inside a glass capillary: liquid at L-V equilibrium **(A)**; the liquid fills in the entire internal volume at Tfill **(B)**; the liquid is forced along an isochore (constant volume) creating higher, positive pressures **(C)**; if the liquid adheres extremely well to the glass capillary walls, at point **(D)** the liquid is experiencing an isotropic, bulk tension (absolute negative pressure metastable state). At Tcav (cavitation temperature) it will collapse at point **(E)**, relaxing back to its stable condition located along its vapor pressure curve at **(F)**. (Reprinted from Silva et al., 2018).

**Figure 11:**
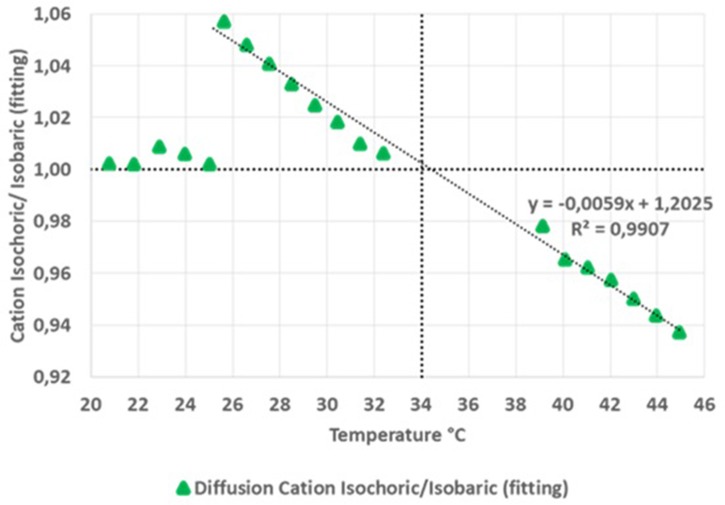
Relative cation's self-diffusion coefficients vs. temperature in positive, negative, and null pressure regimes. (Reprinted from Silva et al., 2018).

Recent work by Silva et al. (2018) has shown that it is possible to use pulsed field gradient NMR spectroscopy to describe the change on the fluid molecular dynamics (transport response functions) of liquid salts under homogeneous negative pressure regimes (down to about −20 MPa). The experiments followed the thermodynamic Berthelot cycle (Figure 10) in order to pre-pressurize the samples and create the conditions to further enter, by cooling the sample, into negative pressure regimes. Negative pressures have been accurately estimated by using experimental values of thermal-pressure coefficients of each liquid at the relevant-temperatures of the corresponding isochore. A marked augment in both anions' and cations' self-diffusion coefficients is observed as one enters the metastable negative pressure region as compared to the saturated liquid conditions. Figure 11 shows the ratio between the cation's diffusion under isochoric conditions and the cation's diffusion under isobaric conditions. The vertical dotted line indicates the filling temperature of the samples, and consequently, data at higher temperatures represent the positive pressure regime while data below this temperature characterize the negative pressure region. It is important to note that for temperatures below 25.5°C (*p* ~ −150 bar), the liquid salt has already cavitated to the L-V condition and, therefore, the ratio in *D*'s was restored to 1.0. The results show a markedly decrease of the self-diffusion coefficient upon applied positive pressure and a steep increase in it for regimes of negative pressure. It is worth to note that a mild volumetric expansion of about 0.6–0.7% (Δ*V*/*V*%) per each Δ*p* = −100 bar of applied negative pressure can be estimated. This contrasts with the increase in the self-diffusion coefficients of about 5% per each Δ*p* = −100 bar, representing an almost 10-fold increase as compared to the volume expansion of the ionic liquid.

## Reversed-Charged Ionic Liquid Pairs

It is well-known that salts based on the halides' anions show much lower melting temperatures than those in which their isoelectronic alkyl metals counterparts are present as cations. For instance, already-known salts based on potassium are not members of the ionic liquids family, whereas many with chloride are.

**Figure 12:**
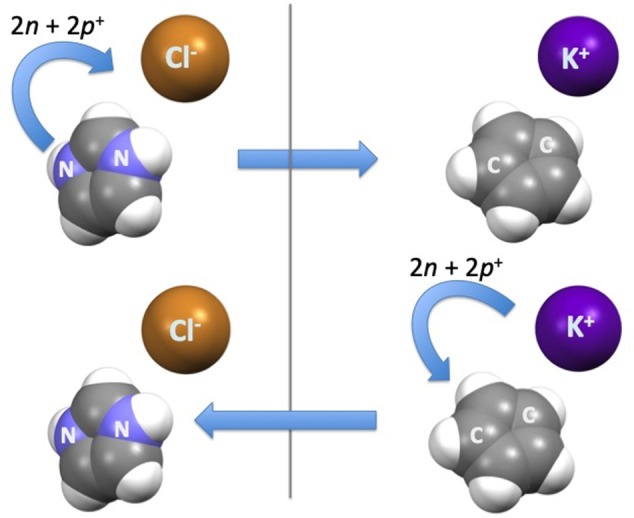
The conceptual transfer of two neutrons and two protons to obtain charge-inverted ionic pairs—the 1-alkyl-3-methylimidazolium chloride and its isoelectronic and isostructural counterpart, the potassium 1-alkyl-3-methylcyclopentadienyl.

It was recently shown (Cruz et al., 2018, 2019) that using alkali metal cations with distinctive 1-alkyl-3-methylcyclopentadienyl anions it is possible to generate ILs. More specifically, if one uses the same isoelectronic concept as we do for alkali metals vs. halides (e.g., K^+^ vs. Cl^−^), it is possible to recognize the 1-alkyl-3-methylcyclopentadienyl anion as the isoelectronic and isostructural counterpart of the 1-alkyl-3-methylimidazolium cation (Figure 12). Conceptually, one simply has to transfer one proton and one neutron from each of the two nitrogen atoms of the imidazolium ring to the nucleus of the chloride anion. Differential scanning calorimetry has been used to show that K[C_4_C_1_Cp] and K[C_6_C_1_Cp] melt without decomposition at around 90°C. Molecular Dynamics (MD) simulations were used to understand the structural differences between these two IL families. The marked structural differences between the K[C_n_C_1_Cp] and [C_n_C_1_im]Cl series is a consequence of the charge-reversion among ion pairs. In the case of these alkali metal based ILs, peculiarities of metal coordination chemistry enables the creation of new structural features. In conclusion, charge-inverted salts can also present low melting temperatures. This fact opens the door for the synthesis of new families of ILs.

### LCST Immiscibility Behavior in IL + Solvent Systems

The liquid-liquid lower critical solution temperature (LCST) type of phase diagram rarely occurs. However, it is an important type of demixing that is the basis of several key applications (Albertsson, 1986). It means that phase separation occurs upon temperature increase, with the system attaining a higher order (as compared to the separated components). The closed-loop phase diagram (a temperature-composition island of immiscibility) is even rarer and appears as a result of a very subtle balance between enthalpic and entropic contributions to the Gibbs energy of a system as temperature is changed. It is characterized by a LCST at a given temperature followed by an upper critical solution temperature (UCST) at a higher temperature.

LCST-type of immiscibility was typically only found in some aqueous or (polymer + solvent) solutions. For the first time, Lachwa et al. (2005) have encountered both LCST and closed-loop type of behavior in binary and *quasi*-binary liquid solutions of alkylmethylimidazolium bistriflimide, [C_n_mim][Ntf_2_], with chloroform or with (chloroform + carbon tetrachloride) mixtures. This study revealed the tunable character of the liquid–liquid phase diagrams involving ILs. Two variables were taken into account, first, in the solutions with chloroform, the number of the carbon atoms in the IL's cation alkyl side chain were varied and secondly the chain length was kept constant at *n* = 5, but the composition of the mixed solvent was altered by adding carbon tetrachloride to chloroform. This work has demonstrated the extreme sensitivity of the phase diagrams upon small changes of two variables: long IL alkyl chain lengths promote better solubility; whereas addition of more CCl_4_ to the CHCl_3_ worsens the solubility.

**Figure 13:**
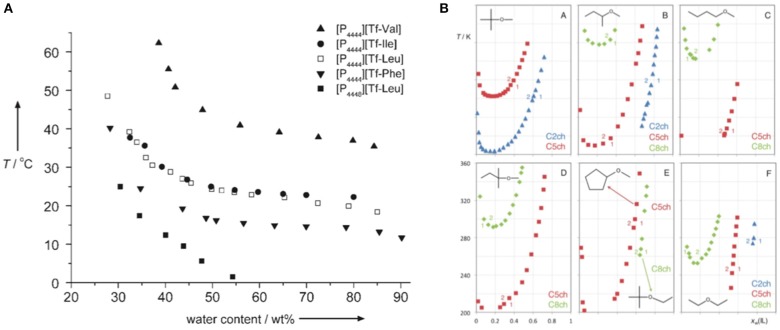
**(A)** Phase-separation temperature vs. water content in mixtures of amino acid based ILs. (Reprinted from Fukumoto and Ohno, 2007 with permission from John Wiley and Sons) **(B)** Cloud point data for different ([N_11 n2OH_][Ntf_2_] + ether) binary mixtures. Each panel depicts a given ether molecule: A = tert-butyl methyl ether; B = sec-butyl methyl ether; C = n-butyl methyl ether; D = tert-amyl methyl ether; E = cyclopentyl methyl ether and tert-butyl ethyl ether; F = diethyl ether. Each marker/color represents a different choline cation in the ionic liquid: blue triangles = [N_1122OH_]^+^ = C2ch; red squares = [N_1152OH_]^+^ = C5ch; green rhombs = [N_1182OH_]^+^ = C8ch. The arabic numerals indicate the number of phases present on each side of the boundaries defined by the cloud point data. (Reprinted from Costa et al., 2013 with permission from Royal Society of Chemistry).

Fukumoto and Ohno (2007) have presented another example of LCST behavior, which was related to the solutions of amino acid-based ILs and water (Figure 13A). The LCST temperatures of the solutions were tuned by changing the length of the alkyl side chains of either the phosphonium cation or the triflate-aminoacid anion. Longer alkyl chains lower the temperature of the phase separation due to enhanced hydrophobicity. The insertion of four methylene groups to one cation's alkyl side chain brings the LCST down from room temperature to the freezing temperature of water. The anion chain addition of only one –CH_2_- group lowers the LCST by about 15°C. Insertion of a phenyl group with a CH_2_ group attached to it also reduces the LCST by about 15°C. The tuning of the LCST behavior has immense potential in extraction and separation processes.

Other cases of LCST behavior within solutions of ILs and polymer as two component systems were demonstrated independently by Ueki and Watanabe (2007) and Lee and Lodge (2011). Alkyl-methylimidazolium bistriflimide ILs, [C_n_mim][NTf_2_], mixed with poly(benzyl-methacrylate) (Ueki and Watanabe, 2007) or poly(n-butyl methacrylate) (Lee and Lodge, 2011) show that the increase of the cation chain length, either in the pure IL or through a mixture of cations with different chains, provokes an opposite effect as compared to similar aqueous solutions. Thereby, longer alkyl chains exhibit better solubility and higher LCST. The authors related the improved solubility to the enhancement of the dispersive forces between the (longer) cation alkyl chains and the polymer chains.

Recently, Costa et al. (2013) have reported for the first time functionalized IL + ether systems exhibiting a LCST behavior. Investigating the phase behavior of binary mixtures of three N-alkyl-N,N-dimethyl-N-hydroxyethylammonium bis(trifluoromethane)sulfonylimide ILs, [N_11n2OH_][Ntf_2_], with nine distinct ethers at atmospheric pressure, they have found that most systems exhibit unusual LCST-type of phase separation (Figure 13B). By increasing the alkyl side chain of the cholinium-derived cation, an enhancement of the mutual solubilities of ILs and ethers is attained. The availability of the oxygen atom of the ether molecule to perform hydrogen bonding with the IL cation and the size/ramification of the alkyl groups of the ether molecule are the key factors that control the solubility of these systems. This LCST behavior is a consequence of the disruption of the H-bond network between the hydroxyl group of the cholinium based cation and the oxygen atom of the ether molecule.

Other works also report systems showing LCST behavior containing mixtures of ILs with polymers, water, supramolecular compounds and other solvents and are summarized in a recent review (Qiao et al., 2017) which highlights their potential applications.

## Concluding Remark

We have highlighted some unexpected features of ionic liquids and ionic liquid-containing systems which are not so well-known of the scientific community.

## Author Contributions

All authors listed have made a substantial, direct and intellectual contribution to the work, and approved it for publication.

### Conflict of Interest Statement

The authors declare that the research was conducted in the absence of any commercial or financial relationships that could be construed as a potential conflict of interest.

## References

[B1] Abraham S. E. Chakrabarati D. Bagchi B. (2007). Energy landscape view of nonideality in binary mixtures. J. Chem. Phys. 126:074501. 10.1063/1.2434967 17328614

[B2] Acree W Jr. Chickos J. S. (2010). Phase transition enthalpy measurements of organic and organometallic compounds. sublimation, vaporization and fusion enthalpies from 1880 to 2010. J. Phys. Chem. Ref. Data 39:043101 10.1063/1.3309507

[B3] Adamová G. Gardas R. L. Rebelo L. P. Robertson A. J. Seddon K. R. (2011). Alkyltrioctylphosphonium chloride ionic liquids: synthesis and physicochemical properties. Dalton Trans. 40, 12750–12764. 10.1039/c1dt10332f 21996935

[B4] Adamova G. Lopes J. N. C. Rebelo L. P. N. Santos L. M. N. B. Seddon K. R. Shimizu K. (2014). The alternation effect in ionic liquid homologous series. Phys. Chem. Chem. Phys. 16, 4033–4038. 10.1039/C3CP54584A 24448218

[B5] Albertsson P. A. (1986). Partitioning of Cell Particles and Macromolecules, 3rd Edn. New York, NY: John Wiley.

[B6] Alvarez Fernandez A. Kouwer P. H. (2016). Key developments in ionic liquid crystals. Int. J. Mol. Sci. 17:731. 10.3390/ijms17050731 27196890 PMC4881553

[B7] Andrzejewska E. Podgorska-Golubska M. Andrzejewski M. (2009). Photoinitiated polymerization in ionic liquids: kinetics and viscosity effects. Polymer 50, 2040–2047. 10.1016/j.polymer.2009.02.034

[B8] Armand M. Endres F. MacFarlane D. R. Ohno H. Scrosati B. (2009). Ionic-liquid materials for the electrochemical challenges of the future. Nat. Mater. 8, 621–629. 10.1038/nmat2448 19629083

[B9] Belchior D. C. V. Sintra T. E. Carvalho P. J. Soromenho M. R. C. Esperança J. M. S. S. Ventura S. P. M.. (2018). Odd-even effect on the formation of aqueous biphasic systems formed by 1-alkyl-3- methylimidazolium chloride ionic liquids and salts. J. Chem. Phys. 148:193842. 10.1063/1.5012020 30283158 PMC6166861

[B10] Belieres J. P. Angell C. A. (2007). Protic ionic liquids: preparation, characterization, and proton free energy level representation. J. Phys. Chem. B 111, 4926–4937. 10.1021/jp067589u 17417896

[B11] Bernardes C. E. S. Canongia Lopes J. N. (2017). Modeling halogen bonds in ionic liquids: a force field for imidazolium and halo-imidazolium derivatives. J. Chem. Theory Comput. 13, 6167–6176. 10.1021/acs.jctc.7b00645 29091432

[B12] Blesic M. Level G. Gilmore B. F. Holbrey J. D. Jacquemin J. Nockemann P. (2017). An introduction to zwitterionic salts. Green Chem.19, 4007–4011. 10.1039/C7GC01523B

[B13] Blokhin A. V. Paulechka Y. U. Strechan A. A. Kabo G. J. (2006). Physicochemical properties, structure, and conformations of 1-butyl-3-methylimidazolium bis(trifluoromethanesulfonyl)imide [C4mim]Ntf2 ionic liquid. J. Phys. Chem. B 112, 4357–4364. 10.1021/jp710872s 18341327

[B14] Brandrup J. Immergut E. H. Grulke E. A. (1999). Polymer Handbook, 4th Edn. New York, NY: John Wiley & Sons, Inc.

[B15] Carvalho P. J. Freire M. G. Marrucho I. M. Queimada A. J. Coutinho J. A. P. (2008). Surface tensions for the 1-Alkyl-3-methylimidazolium Bis(trifluoromethylsulfonyl)imide ionic liquids. J. Chem. Eng. Data 53, 1346–1350. 10.1021/je800069z

[B16] Cavallo G. Bruce D. W. Terraneo G. Resnati G. Metrangolo P. (2018). From molecules to materials: engineering new ionic liquid crystals through halogen bonding. J. Vis. Exp. 133:e55636 10.3791/55636 PMC5933245 29630052

[B17] Cerdeirina C. A. Tovar C. A. Gonzalez-Salgado D. Carballo E. Roman,í L. (2001). Isobaric thermal expansivity and thermophysical characterization of liquids and liquid mixtures. Phys. Chem. Chem. Phys. 3, 5230–5236. 10.1039/b104891k

[B18] Chatel G. Pereira J. F. B. Debbeti V. Wang H. Rogers R. D. (2014). Mixing ionic liquids – “simple mixtures” or “double salts”? Green Chem. 16, 2051–2083. 10.1039/c3gc41389f

[B19] Costa A. J. L. Soromenho M. R. C. Shimizu K. Esperança J. M. S. S. Lopes J. N. C. Rebelo L. P. N. (2013). Unusual LCST-type behaviour found in binary mixtures of choline-based ionic liquids with ethers. RSC Adv. 3, 10262–10271. 10.1039/c3ra40327k

[B20] Cruz T. F. C. Shimizu K. Esperança J. M. S. S. André V. Duarte M. T. Rebelo L. P. N. (2019). ILs in wonderland: from electrostatics to coordination chemistry. J Phys. Chem. C 123, 5804–5811. 10.1021/acs.jpcc.9b00987

[B21] Cruz T. F. C. Shimizu K. Esperança J. M. S. S. Rebelo L. P. N. Gomes P. T. Canongia Lopes J. N. (2018). ILs through the looking glass: electrostatics and structure probed using charge-inverted ionic liquid pairs. Faraday Discuss. 206, 203–218. 10.1039/C7FD00139H 28930331

[B22] de Ferro A. M. Reis P. M. Soromenho M. R. C. Bernardes C. E. S. Shimizu K. Freitas A. A.. (2018). Designing the ammonium cation to achieve a higher hydrophilicity of bistriflimide-based ionic liquids. Phys. Chem. Chem. Phys. 20, 19307–19313. 10.1039/C8CP03398F 29900442

[B23] Domalski E. S. Hearing E. D. (1996). Heat capacities and entropies of organic compounds in the condensed phase volume III. J. Phys. Chem. Ref. Data 25, 1–523. 10.1063/1.555985

[B24] Dzida M. Musiał M. Zorebski E. Zorebski M. Jacquemin J. Goodrich P. (2018). Comparative study of effect of alkyl chain length on thermophysical characteristics of five N-alkylpyridinium bis(trifluoromethylsulfonyl)imides with selected imidazolium- based ionic liquids. J. Mol. Liq. 271, 401–412. 10.1016/j.molliq.2019.01.022

[B25] Esperança J. M. S. S. Canongia Lopes J. N. Tariq M. Santos L. M. N. B. F. Magee J. W. Rebelo L. P. N. (2010). Volatility of aprotic ionic liquids—a review. J. Chem. Eng. Data 55, 3–12. 10.1021/je900458w

[B26] Ferreira A. I. M. C. L. Rodrigues A. Villas M. Tojo E. Rebelo L. P. N. Santos L. M. N. B. F. (2019). Crystallization and glass-forming ability of ionic liquids: novel insights into their thermal behavior. ACS Sust. Chem. Eng. 7, 2989–2997. 10.1021/acssuschemeng.8b04343

[B27] Fujita K. Kajiyama M. Liu Y. Nakamura N. Ohno H. (2016). Hydrated ionic liquids as a liquid chaperon for refolding of aggregated recombinant protein expressed in *Escherichia coli*. Chem. Commun. 13491–13494. 10.1039/C6CC06999A 27801474

[B28] Fukumoto K. Ohno H. (2007). LCST-type phase changes of a mixture of water and ionic liquids derived from amino acids. Angew. Chem. Int. Ed. 46, 1852–1855. 10.1002/anie.200604402 17274096

[B29] Gardas R. L. Coutinho J. A. P. (2008). A group contribution method for heat capacity estimation of ionic liquids. Ind. Eng. Chem. Res. 47, 5751–5757. 10.1021/ie800330v

[B30] Goḿez E. Calvar N. Domínguez Á. Macedo E. A. (2013). Thermal analysis and heat capacities of 1-Alkyl-3-methylimidazolium ionic liquids with NTf2-, TFO-, and DCA- anions. Ind. Eng. Chem. Res. 52, 2103–2110. 10.1021/ie3012193

[B31] Goossens K. Lava K. Bielawski C. W. Binnemans K. (2016). Ionic liquid crystals: versatile materials. Chem. Rev.116, 4643–4807. 10.1021/cr400334b 27088310

[B32] Haberler M. Schröder C. Steinhauser O. (2012). Hydrated ionic liquids with and without solute: the influence of water content and protein solutes. J. Chem. Theory Comput. 8, 3911–3928. 10.1021/ct300191s 26593031

[B33] Haddad J. Pontoni D. Murphy B. M. Festersen S. Runge B. Magnussen O. M. (2018). Surface structure evolution in a homologous series of ionic liquids. *Proc. Nat. Acad. Sci*. U. S. A. 115, E1100–E1107. 10.1073/pnas.1716418115 PMC5819424 29358372

[B34] Hallett J. P. Welton T. (2011). Room-temperature ionic liquids: solvents for synthesis and catalysis. 2. Chem. Rev. 111, 3508–3576. 10.1021/cr1003248 21469639

[B35] Hayes R. Warr G. G. Atkin R. (2015). Structure and nanostructure in ionic liquids. Chem. Rev. 115, 6357–6426. 10.1021/cr500411q 26028184

[B36] Hunt P. A. Ashworth C. R. Matthews R. P. (2015). Hydrogen bonding in ionic liquids. Chem. Soc. Rev. 44, 1257–1288. 10.1039/C4CS00278D 25582457

[B37] Kar M. Plechkova N. V. Seddon K. R. Pringle J. M. MacFarlane D. R. (2019). Ionic liquids – further progress on the fundamental issues. Aust. J. Chem. 72, 3–10. 10.1071/CH18541

[B38] Kolbeck C. Lehmann J. Lovelock K. R. Cremer T. Paape N. Wasserscheid P.. (2010). Density and surface tension of ionic liquids. J. Phys. Chem. B 114, 17025–17036. 10.1021/jp1068413 21141903

[B39] Kouris S. Panaylotou C. (1989). Dynamic viscosity of mixtures of benzene, ethanol and n-heptane at 298.15 K. J. Chem. Eng. Data 34, 200–203. 10.1021/je00056a016

[B40] Lachwa J. Szydlowski J. Najdanovic-Visak V. Rebelo L. P. Seddon K. R. da Ponte M. N.. (2005). Evidence for lower critical solution behavior in ionic liquid solutions. J. Am. Chem. Soc. 127, 6542–6543. 10.1021/ja0510257 15869269

[B41] Laesecke A. Freund M. Morrison E. (2007). Molecular interactions in nonpolar+polar binary mixtures: measurements of N-hexadecane+butyl benzoate, in AIChE Annu Meeting (Salt Lake City, UT). 384b.

[B42] Leal J. P. da Piedade M. E. Lopes J. N. Tomaszowska A. A. Esperança J. M. Rebelo L. P. N.. (2009). Bridging the gap between ionic liquids and molten salts: group 1 metal salts of the bistriflamide anion in the gas phase. J. Phys. Chem. B 113, 3491–3498. 10.1021/jp811039b 19278265

[B43] Lee H. N. Lodge T. P. (2011). Poly(n-butyl methacrylate) in ionic liquids with tunable lower critical solution temperatures (LCST). J. Phys. Chem. B 115, 1971–1977. 10.1021/jp1106058 21322624

[B44] Leys J. Tripathi C. S. Glorieux C. Zahn S. Kirchner B. Longuemart S.. (2014). Electrical conductivity and glass formation in nitrile-functionalized pyrrolidinium bis(trifluoromethylsulfonyl)imide ionic liquids: chain length and odd–even effects of the alkyl spacer between the pyrrolidinium ring and the nitrile group. Phys. Chem. Chem. Phys. 16, 10548–10557. 10.1039/c4cp00259h 24740743

[B45] Lima T. A. Faria L. F. O. Paschoal V. H. Ribeiro M. C. C. (2018). Glass transition and melting lines of an ionic liquid. J. Chem. Phys. 148:171101. 10.1063/1.5030083 29739222

[B46] Lodeiro L. Contreras R. Ormazábal-Toledo R. (2018). How meaningful is the halogen bonding in 1-ethyl-3-methyl imidazolium-based ionic liquids for Co2 capture? J. Phys. Chem. B 122, 7907–7914. 10.1021/acs.jpcb.8b04990 30036060

[B47] Lopes J. N. Gomes M. F. Pádua A. A. (2006). Nonpolar, polar, and associating solutes in ionic liquids. J. Phys. Chem. B 110, 16816–16818. 10.1021/jp063603r 16927967

[B48] Lopes J. N. Pádua A. A. (2006). Nanostructural organization in ionic liquids. J. Phys. Chem. B 110, 3330–3335. 10.1021/jp056006y 16494347

[B49] Luís A. Shimizu K. Araújo J. M. M. Carvalho P. J. Lopes-da-Silva J. A. Lopes J. N.. (2016). Influence of nanosegregation on the surface tension of fluorinated ionic liquids. Langmuir 32, 6130–6139. 10.1021/acs.langmuir.6b00209 27218210 PMC5325320

[B50] Marsh K. N. Boxall J. A. Lichtenthaler R. (2004). Room temperature ionic liquids and their mixtures—a review. Fluid Phase Equilib. 219, 93–98. 10.1016/j.fluid.2004.02.003

[B51] Mirkhani S. A. Gharagheizi F. Ilani-Kashkouli P. Farahani N. (2012). Determination of the glass transition temperature of ionic liquids: a molecular approach. Thermochimica Acta 543, 88–95. 10.1016/j.tca.2012.05.009

[B52] Navia P. Troncoso J. Romaní L. (2010a). Isobaric thermal expansivity for ionic liquids with a common cation as a function of temperature and pressure. J. Chem. Eng. Data 55, 590–594. 10.1021/je900407u

[B53] Navia P. Troncoso J. Romani L. (2010b). Dependence against temperature and pressure of the isobaric thermal expansivity of room temperature ionic liquids. J. Chem. Eng. Data 55, 595–599. 10.1021/je900482x

[B54] Niedermeyer H. Hallett J. P. Villar-Garcia I. J. Hunt P. A. Welton T. (2012). Mixtures of ionic liquids. Chem. Soc. Rev. 41, 7780–7802. 10.1039/c2cs35177c 22890419

[B55] Nieto de Castro C. A. Langa E. Morais A. L. Lopes M. L. M. Lourenco M. J. V. Santos F. J. V. (2010). Studies on the density, heat capacity, surface tension and infinite dilution diffusion with the ionic liquids [C4mim][NTf2], [C4mim][dca], [C2mim][EtOSO3], and [Aliquat][dca]. Fluid Phase Equilib. 294, 157–179. 10.1016/j.fluid.2010.03.010

[B56] Ohno H. Yoshizawa-Fujita M. Kohno Y. (2018). Design and properties of functional zwitterions derived from ionic liquids. Phys. Chem. Chem. Phys. 20, 10978–10991. 10.1039/C7CP08592C 29620779

[B57] Pádua A. A. Costa Gomes M. F. Lopes J. N. (2007). Molecular solutes in ionic liquids: a structural perspective. Acc. Chem. Res. 40, 1087–1096. 10.1021/ar700050q 17661440

[B58] Papanastaaiou G. E. Ziogas I. I. (1991). Physical behaviour of some reaction media. Density, viscosity, dielectric constant, and refractive index change of ethanol-cyclohexane mixtures at several temperatures. J. Chem. Eng. Data 36, 46–51. 10.1021/je00001a014

[B59] Paulechka Y. U. Kabo A. G. Blokhin A. V. Kabo G. J. Shevelyova M. P. (2010). Heat capacity of ionic liquids: experimental determination and correlations with molar volume. J. Chem. Eng. Data 55, 2719–2724. 10.1021/je900974u

[B60] Pereiro A. B. Araújo J. M. M. Teixeira F. S. Marrucho I. M. Piñeiro M. M. Rebelo L. P. N. (2015). Aggregation behavior and total miscibility of fluorinated ionic liquids in water. Langmuir 31, 1283–1295. 10.1021/la503961h 25580898

[B61] Pereiro A. B. Pastoriza-Gallego M. J. Shimizu K. Marrucho I. M. Canongia Lopes J. N. Piñeiro M. M.. (2013). On the formation of a third, nanostructured domain in ionic liquids. J. Phys. Chem. B 117, 10826–10833. 10.1021/jp402300c 23964834

[B62] Plechkova N. V. Seddon K. R. (2008). Applications of ionic liquids in the chemical industry. Chem. Soc. Rev. 37, 123–150. 10.1039/B006677J 18197338

[B63] Podgoršek A. Jacquemin J. Pádua A. A. H. Costa Gomes M. F. (2016). Mixing enthalpy for binary mixtures containing ionic liquids. Chem. Rev. 116, 6075–6106. 10.1021/acs.chemrev.5b00379 27144455

[B64] Qiao Y. Ma W. Theyssen N. Chen C. Hou Z. (2017). Temperature-responsive ionic liquids: fundamental behaviors and catalytic applications. Chem. Rev. 117, 6881–6928. 10.1021/acs.chemrev.6b00652 28358505

[B65] Qunfang L. Yu-Chun H. (1999). Correlation of viscosity of binary liquid mixtures. Fluid Phase Equilib. 154, 153–163. 10.1016/S0378-3812(98)00415-4

[B66] Rahman M. H. Senapati S. (2018). Water clathrates in nanostructural organization of hydrated ionic liquids manifest in peculiar density trend. J. Phys. Chem. B. 123, 1592–1601. 10.1021/acs.jpcb.8b08586 30475622

[B67] Rebelo L. P. N. Najdanovic-Visak V. Visak Z. P. Nunes da Ponte M. Szydlowski J. Cerdeirina C. A. (2004). A detailed thermodynamic analysis of [C4mim][BF4] + water as a case study to model ionic liquid aqueous solutions. Green Chem. 6, 369–381. 10.1039/B400374H

[B68] Rocha M. A. Coutinho J. A. Santos L. M. (2012). Cation symmetry effect on the volatility of ionic liquids. J. Phys. Chem. B 116, 10922–10927. 10.1021/jp306937f 22873766

[B69] Rocha M. A. Coutinho J. A. Santos L. M. (2014). Vapor pressures of 1,3-dialkylimidazolium bis(trifluoromethylsulfonyl)imide ionic liquids with long alkyl chains. J. Chem. Phys. 141:134502. 10.1063/1.4896704 25296816

[B70] Rocha M. A. Neves C. M. Freire M. G. Russina O. Triolo A. Coutinho J. A.. (2013). Alkylimidazolium based ionic liquids: impact of cation symmetry on their nanoscale structural organization. J. Phys. Chem. B 117, 10889–10897. 10.1021/jp406374a 23915346

[B71] Rodrigues A. S. Santos L. M. (2016). Nanostructuration effect on the thermal behavior of ionic liquids. ChemPhysChem 17, 1512–1517. 10.1002/cphc.201501128 26888172

[B72] Rodríguez H. Brennecke J. F. (2006). Temperature and composition dependence of the density and viscosity of binary mixtures of water + ionic liquid. J. Chem. Eng. Data 51, 2145–2155. 10.1021/je0602824

[B73] Saccone M. Palacio F. F. Cavallo G. Dichiarante V. Virkki M. Terraneo G.. (2017). Photoresponsive ionic liquid crystals assembled via halogen bond: en route towards light-controllable ion transporters. Faraday Discuss. 203, 407–422. 10.1039/C7FD00120G 28725887

[B74] Sanmamed Y. A. Gonzalez-Salgado D. Troncoso J. Cerdeirina C. A. Romaní L. (2007). Viscosity-induced errors in the density determination of room temperature ionic liquids using vibrating-tube densitometry. Fluid Phase Equilib. 252, 96–102. 10.1016/j.fluid.2006.12.016

[B75] Santos L. M. N. B. F. Canongia Lopes J. N. Coutinho J. A. P. Esperança J. M. S. S. Gomes L. R. Marrucho I. M.. (2007). Ionic liquids: first direct determination of their cohesive energy. J. Am. Chem. Soc. 129, 284–285. 10.1021/ja067427b 17212402

[B76] Serra P. B. P. Ribeiro F. M. S. Rocha M. A. A. Fulem M. RuŽicka K. Coutinho J. A. P. (2017). Solid-liquid equilibrium and heat capacity trend in the alkylimidazolium PF6 series. J. Mol. Liq. 248, 678–687. 10.1016/j.molliq.2017.10.042

[B77] Shimizu K. Heller B. S. J. Maier F. Steinrück H. P. Lopes J. N. (2018). Probing the surface tension of ionic liquids using the langmuir principle. Langmuir 34, 4408–4416. 10.1021/acs.langmuir.7b04237 29485882 PMC5911805

[B78] Shimizu K. Tariq M. Costa Gomes M. F. Rebelo L. P. N. Lopes J. N. C. (2010). Assessing the dispersive and electrostatic components of the cohesive energy of ionic liquids using molecular dynamics simulations and molar refraction data. J. Phys. Chem. B 114, 5831–5834. 10.1021/jp101910c 20392094

[B79] Silva W. Veiga H. I. M. Tariq M. Cabrita E. J. Esperança J. M. S. S. Canongia Lopes J. N. (2018). Negative pressure regimes in ionic liquids: structure and interactions in stretched liquids as probed by NMR. ECS Trans. 86, 141–147. 10.1149/08614.0141ecst

[B80] Singh A. P. Gardas R. L. Senapati S. (2015). Divergent trend in density versus viscosity of ionic liquid/water mixtures: a molecular view from guanidinium ionic liquids. Phys. Chem. Chem. Phys. 17, 25037–25048. 10.1039/C5CP02841H 26347332

[B81] Singh A. P. Gardas R. L. Senapati S. (2017). How water manifests the structural regimes in ionic liquids. Soft Matter 13, 2348–2361. 10.1039/C6SM02539K 28275768

[B82] Srinivas G. Mukherjee A. Bagchi B. (2001). Nonideality in the composition dependence of viscosity in binary mixtures. J. Chem. Phys. 114:6220 10.1063/1.1354166

[B83] Strechan A. A. Kabo A. G. Paulechka Y. U. Blokhin A. V. Kabo G. J. Shaplov A. S. (2008). Thermochemical properties of 1-butyl-3-methylimidazolium nitrate. Thermochimica Acta 474, 25–31. 10.1016/j.tca.2008.05.002

[B84] Tao R. Gurung E. Cetin M. M. Mayer M. F. Quitevis E. L. Simon S. L. (2017). Fragility of ionic liquids measured by Flash differential scanning calorimetry. Thermochimica Acta 654, 121–129. 10.1016/j.tca.2017.05.008

[B85] Tariq M. Forte P. A. S. Gomes M. F. C. Lopes J. N. C. Rebelo L. P. N. (2009). Densities and refractive indices of imidazolium- and phosphonium-based ionic liquids: effect of temperature, alkyl chain length, and anion. J. Chem. Thermodynamics 41, 790–798. 10.1016/j.jct.2009.01.012

[B86] Tariq M. Freire M. G. Saramago B. Lopes J. N. Coutinho J. A. P. Rebelo L. P. N. (2012). Surface tension of ionic liquids and ionic liquid solutions. Chem. Soc. Rev. 41, 829–868. 10.1039/C1CS15146K 21811714

[B87] Tariq M. Serro A. P. Mata J. L. Saramago B. Esperança J. M. S. S. Lopes J. N. C. (2010). High-temperature surface tension and density measurements of 1-alkyl-3-methylimidazolium bistriflamide ionic liquids. Fluid Phase Equilib. 294, 131–138. 10.1016/j.fluid.2010.02.020

[B88] Tariq M. Shimizu K. Esperança J. M. S. S. Lopes J. N. C. Rebelo L. P. N. (2015). Viscosity minima in binary mixtures of ionic liquids + molecular solvents. Phys. Chem. Chem. Phys. 17, 13480–13494. 10.1039/C5CP01563D 25933136

[B89] Triolo A. Russina O. Bleif H. J. Di Cola E. (2007). Nanoscale segregation in room temperature ionic liquids. J. Phys. Chem. B 111, 4641–4644. 10.1021/jp067705t 17388414

[B90] Trivedi S. Pandey S. (2011). Interactions within a [IonicLiquid+ Poly(ethylene glycol)] mixture revealed by temperature-dependent synergistic dynamic viscosity and probe-reported microviscosity. J. Phys. Chem. B 115, 7405–7416. 10.1021/jp203079p 21591689

[B91] Troncoso J. Cerdeirina C. A. Navia P. Sanmamed Y. A. Gonzalez-Salgado D. Romani L. (2010). Unusual behavior of the thermodynamic response functions of ionic liquids. J. Phys. Chem. Lett. 1, 211–214. 10.1021/jz900049g

[B92] Ueki T. Watanabe M. (2007). Lower critical solution temperature behavior of linear polymers in ionic liquids and the corresponding volume phase transition of polymer gels. Langmuir 23, 988–990. 10.1021/la062986h 17241001

[B93] Valderrama J. O. Campusano R. A. Rojas R. E. (2017). Glass transition temperature of ionic liquids using molecular descriptors and artificial neural networks. C. R. Chimie 20, 573–584. 10.1016/j.crci.2016.11.009

[B94] Visak Z. P. Rebelo L. P. N. Szydlowski J. (2002). Achieving absolute negative pressures in liquids: precipitation phenomena in solution. J. Chem Educ. 79, 869–873. 10.1021/ed079p869

[B95] Visak Z. P. Rebelo L. P. N. Szydlowski J. (2003). The “hidden” phase diagram of water+3-methylpyridine at large absolute negative pressures. J. Phys. Chem. B 107, 9837–9846. 10.1021/jp0223206

[B96] Welton T. (1999). Room-temperature ionic liquids: solvents for synthesis and catalysis. Chem. Rev. 99, 2071–2084. 10.1021/cr980032t 11849019

[B97] Welton T. (2018). Ionic liquids: a brief history. Biophys. Rev. 10, 691–706. 10.1007/s12551-018-0419-2 29700779 PMC5988633

[B98] Wu B. Kuroda K. Takahashi K. Castner E. W. (2018). Structural analysis of zwitterionic liquids vs. homologous ionic liquids. J. Chem. Phys.148:193807. 10.1063/1.5010983 30307210

[B99] Xu W. Angell C. A. (2003). Solvent-free electrolytes with aqueous solution–like conductivities. Science 302, 422–425. 10.1126/science.1090287 14564002

[B100] Yang K. Cai Z. Tyagi M. Feygenson M. Neuefeind J. C. Moore J. S. (2016). Odd–even structural sensitivity on dynamics in network-forming ionic liquids. Chem. Mater. 28, 3227–3233. 10.1021/acs.chemmater.6b01429

[B101] Yaws C. L. (2009). Thermophysical Properties of Chemicals and Hydrocarbons. Elsevier.

[B102] Zabransky M. Ruzicka V Jr. Majer V. (1990). Heat capacities of organic compounds in the liquid state I. C1 to C18 1-alkanols. J. Phys. Chem. Ref. Data 19, 719–762. 10.1063/1.555860

